# Frequency-dependent modulation of cerebellar excitability during the application of non-invasive alternating current stimulation

**DOI:** 10.1016/j.brs.2021.01.007

**Published:** 2021

**Authors:** Danny Spampinato, Esin Avci, John Rothwell, Lorenzo Rocchi

**Affiliations:** a Department of Clinical and Movement Neurosciences, Institute of Neurology, University College London, United Kingdom; b Non-invasive Brain Stimulation Unit, IRCCS Santa Lucia Foundation, Via Ardeatina 306/354, 00142, Rome, Italy; c Department of Sport and Sport Science, Institute of Biology, University of Freiburg, Germany

**Keywords:** Cerebellum, tACS, TMS, Neurophysiology

## Abstract

**Background:**

it is well-known that the cerebellum is critical for the integrity of motor and cognitive actions. Applying non-invasive brain stimulation techniques over this region results in neurophysiological and behavioural changes, which have been associated with the modulation of cerebellar-cerebral cortex connectivity. Here, we investigated whether online application of cerebellar transcranial alternating current stimulation (tACS) results in changes to this pathway.

**Methods:**

thirteen healthy individuals participated in two sessions of cerebellar tACS delivered at different frequencies (5Hz and 50Hz). We used transcranial magnetic stimulation to measure cerebellar-motor cortex (M1) inhibition (CBI), short-intracortical inhibition (SICI) and short-afferent inhibition (SAI) before, during and after the application of tACS.

**Results:**

we found that CBI was specifically strengthened during the application of 5Hz cerebellar tACS. No changes were detected immediately following the application of 5Hz stimulation, nor at any time point with 50Hz stimulation. We also found no changes to M1 intracortical circuits (i.e. SICI) or sensorimotor interaction (i.e. SAI), indicating that the effects of 5Hz tACS over the cerebellum are site-specific.

**Conclusions:**

cerebellar tACS can modulate cerebellar excitability in a time- and frequency-dependent manner. Additionally, cerebellar tACS does not appear to induce any long-lasting effects (i.e. plasticity), suggesting that stimulation enhances oscillations within the cerebellum only throughout the stimulation period. As such, cerebellar tACS may have significant implications for diseases manifesting with abnormal cerebellar oscillatory activity and also for future behavioural studies.

## Highlights

• Cerebellar tACS increases the inhibitory tone that the cerebellum exerts over M1 (CBI).
• CBI changes were found only during the online application of 5Hz tACS and not immediately following stimulation.
• The effects are specific to the cerebellum, as no changes were found in intracortical measures (e.g. SICI and SAI).

## Introduction

The cerebellum plays a vital role in the several daily actions humans perform, including adapting and fine-tuning of movements [1], as well as in performing behaviours with cognitive demands [2,3]. For instance, individuals with cerebellar pathology typically lose their ability to perform smooth and coordinated actions, a deficit often compounded with moderate cognitive impairments, such as impaired working memory [[4], [5], [6]]. Thus, developing novel interventions to modulate cerebellar activity is critical for providing further insights into cerebellar function, and may also serve as a potential rehabilitation strategy for patients with cerebellar diseases affecting both motor and cognitive domains.

Our capacity to perform a wide range of cognitive and motor actions rely on the vast connections between the cerebellum and various brain regions, including prefrontal, parietal and motor areas (e.g. primary motor (M1) and premotor cortex) [7,8]. In particular, cerebellar-thalamic cortical pathways between the cerebellum and M1 have been extensively studied with paired-pulse transcranial magnetic stimulation (TMS). To assess cerebellar-M1 connectivity, a conditioning stimulus is administered over one cerebellar hemisphere 5–7 ms before stimulating M1, which results in reduced motor evoked potential (MEP) amplitudes [9], [10], [62]. This response, termed cerebellar-M1 inhibition (CBI), has been interpreted to reflect the activation of cerebellar Purkinje cells that hinders the excitatory drive of cerebellar-thalamic pathways (Celnik 2015). Importantly, CBI changes following motor learning or plasticity inducing protocols are thought to represent changes in cerebellar excitability independent of M1 activity [[11], [12], [13]].

Modulation of CBI has been previously achieved with non-invasive brain stimulation techniques such as repetitive transcranial magnetic stimulation (rTMS) and transcranial direct current stimulation (tDCS) [[14], [15], [16], [17], [18]]. However, there are inconsistencies in terms of both the magnitude and direction of effects with these approaches [19,20]. This is most likely due to the substantial inter-individual variability and lack of neural predictors in response to brain stimulation. In part, this is explained by the limited range in which stimulation can target the cerebellum [21], including where stimulation should be applied (e.g. location of figure-eight TMS coil) and which montage should be selected (e.g. polar-dependency of tDCS).

Transcranial alternating current stimulation (tACS) has emerged as a promising alternative technique that can alter healthy individuals’ motor and cognitive state [22,23]. Recent work has argued that tACS can enhance cortical oscillations by entraining brain rhythms to desired frequencies underneath the stimulated site [24,25]. In other words, experimenters may set stimulation parameters to modulate physiologically relevant brain oscillations. One study has shown changes to occur in cerebellar excitability following tACS application (i.e. offline measure) [61]; however, it remains unknown how cerebellar tACS affects the brain during stimulation. It is important to understand the physiological changes that occur during stimulation as the main behavioural effects of tACS have been documented online [[26], [27], [28], [29], [30]]. For instance, cerebellar tACS can entrain the phase of ongoing limb oscillation in essential tremor and parkinsonian tremor [31] and can modulate gait rhythm in healthy individuals [32].

Here, we investigated whether applying cerebellar tACS at specific frequencies can modulate online cerebellar activity. To do this, we applied cerebellar tACS at two distinct frequencies to determine whether it can modulate CBI and intracortical circuits within M1. Although the oscillatory activities in the cerebellar cortex cover a wide range of frequencies, we selected two of them based on the resonance of cerebellar granule cells (theta-band; 5Hz) and basal firing patterns of Purkinje cells (gamma-band; 50Hz). Considering that previous work has demonstrated that cerebellar granule cells and Golgi cells have a preferential response frequency in the theta-band [33], [34], [35], [60]; and that TMS theta-burst modulates cerebellar excitability in humans [15,36,37], we predicted that increasing their oscillatory activity via theta-frequency stimulation would elicit stronger CBI responses. In other words, we expected CBI changes to most prominently occur during 5Hz cerebellar tACS.

## Methods

### Participants

Thirteen right-handed healthy individuals (5 males and 8 females; mean age ± SD: 26.7 ± 5.26; range: 21–37 years) participated in the study. All participants reported no contraindications to TMS [38] and had normal (or corrected to normal) visual acuity. The study was approved by the ethics committee of University College London and all experimental procedures were performed in accordance with the Declaration of Helsinki. All participants gave their informed consent for the experimental procedures.

### Experimental setup

**Fig. 1:**
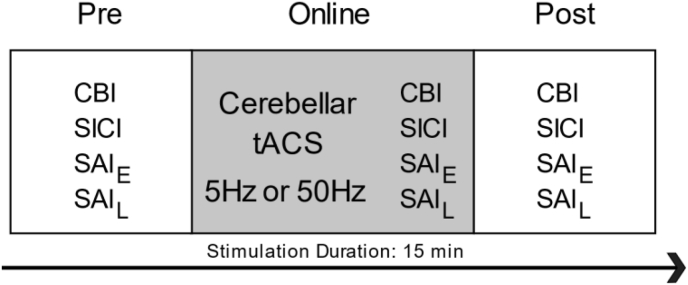
Experimental procedure. All individuals participated in a cross-over experimental design in which the frequency of cerebellar tACS (5Hz and 50Hz) was randomized between sessions. We first measured baseline physiological responses (pre) of cerebellar excitability (CBI), short-intracortical inhibition (SICI) and varying (early and late) somatosensory afferent inputs (SAI_E_, SAI_L_). Cerebellar tACS was then administered over the right cerebellar cortex for 15 min. We repeated the same baseline measures during (online) and immediately following the end of stimulation (post).

All individuals participated in two randomly ordered experimental sessions separated by at least 48 h to evaluate the effect of cerebellar tACS applied with different frequencies. For the first session, the order of TMS physiological assessments was selected at random. This randomly selected order was then repeated in the second session (Fig. 1).

### Cerebellar transcranial alternating current stimulation (tACS)

Alternating current stimulation was delivered (NeuroConn GmbH, Ilmenau, Germany) through two sponge electrodes (5 × 5 cm) soaked in saline solution. One electrode was centered 1 cm below and 3 cm lateral to the inion over the right cerebellar cortex. The other was placed over the right buccinator muscle. Cerebellar tACS was administered with a current intensity of 1.5 mA and consisted of 4500 cycles at 5 Hz and 45000 cycles at 50 Hz (i.e. 15-minute duration). No offset, fade in/out, phase and triggering cycle were set. Two tACS frequencies (5Hz and 50Hz) were administered in different sessions, separated by at least 48 h. The order of these two sessions was randomized. For each session, participants were asked to rate the level of stimulation intensity, itchiness and pain on a scale from 0 (none) to 4 (high). Across sessions, we found no differences in the reports of stimulation intensity (5Hz: 1.54 ± 0.22; 50Hz = 1.69 ± 0.32; p = 0.502), itchy sensations (5Hz: 1.23 ± 0.29; 50Hz = 1.15 ± 0.33; p = 0.673) or pain (5Hz: 0.46 ± 0.19; 50Hz = 0.54 ± 0.19; p = 0.753). Additionally, there were no reports of visual percepts (i.e. flickering lights) during or following stimulation.

### Electromyographic (EMG) recordings

Surface EMG was recorded through pairs of disposable electrodes placed over the right first dorsal interosseous (FDI) muscle (Whitesensor 40713, Ambu®, Denmark). The reference electrode was placed on the proximal phalanx of the index finger and the ground electrode was placed on the right wrist. All EMG signals were amplified with a gain of 1000 (Digitimer 360, Digitimer, UK), band-pass filtered between 20 and 2000 Hz and acquired at 5 kHz sampling rate (Micro 1401 AD Converter; Cambridge Electronics Design (CED), Cambridge, UK). EMG activities were constantly monitored on a computer screen to ensure the muscle was relaxed throughout the duration of the experiments. Data were stored and analyzed offline with Signal 7.04 software (CED, Cambridge, UK).

### Transcranial magnetic stimulation (TMS)

All TMS pulses were administered with a Magstim 200^2^ monophasic stimulator (Magstim, Whitland, Dyfed, UK). To stimulate M1, we used a standard figure-of-eight coil (80 mm diameter). The coil was placed tangentially to the scalp, angled 45° to the mid-sagittal plane such that a posterior-to-anterior current was induced to the brain. The TMS hotspot was located by finding the spot over the left M1 that elicited the largest MEPs in the contralateral FDI. Participants wore a tight fitted-cap with the marked hotspot to provide visual reference of the stimulation site. Once the hotspot was found, the resting motor threshold (RMT) was determined by adjusting the TMS output to the lowest intensity that evoked MEPs of 50 μV in 5 of 10 trials at rest [39]. We next found the stimulator intensity required to elicit a MEP of approximately 1 mV amplitude. This intensity was used for all suprathreshold TMS pulses applied over M1. The inter-trial interval between TMS pulses was randomized between 4 and 6 s.

### Cerebellar-M1 connectivity (CBI)

Cerebellar TMS was performed with a Magstim double-cone coil (110 mm diameter), placed over the right cerebellar hemisphere, centered 3 cm lateral to the inion with an upward current induced to the brain [62]. The coil was positioned in this manner to ensure optimal stimulation of the cerebellum without directly activating the spinal cord [9,40]. For all measures requiring cerebellar stimulation, we set the TMS intensity to 60% of maximum stimulator output (MSO), as this value does not active the brainstem and is easily tolerated by participants [16,21].

We assessed connectivity between the cerebellum and M1 (termed CBI) by using an established paired-pulse stimulation protocol [62]. For each CBI assessment, we recorded 30 TMS test stimuli (TS) over the left M1 that were set at intensity to elicit an MEP ∼1 mV. In half of these trials, selected randomly, a TMS conditioning stimulus (CS) was delivered over the right cerebellum 5 ms prior to the TS. Thus, a total of 15 TS and 15 CS + TS pulses were administered. CBI was calculated as the ratio of the mean MEP amplitude in the CS + TS relative to TS.

### Short-interval intracortical inhibition (SICI)

To assess local inhibitory circuits within M1, SICI was measured using a paired-pulse protocol [41,42]. Here, an interstimulus interval of 3 ms was selected between a subthreshold CS and a suprathreshold TS. The CS intensity was set at 80% RMT and the suprathreshold TS intensity was set to elicit a ∼1 mV MEP response. Similar to CBI, we measured SICI as a ratio of 15 CS + TS over 15 TS MEP responses.

### Short-afferent inhibition

Previous work suggested that late-arriving peripheral sensory inputs to M1 (i.e. 25 ms or greater from distal hand muscle) might follow a trans-cerebellar route [17]. Therefore, we investigated whether cerebellar tACS interferes with this processing, compared to early arriving inputs. To do so, we measured SAI by using an established protocol [43,44]. Conditioning stimuli consisted of square-wave electrical pulses of 200 μs duration applied to the right ulnar nerve through a constant current stimulator (Digitimer, DS7A, Hertfordshire, UK). Stimulation intensity was adjusted to elicit a 0.2 mV M-wave in the right FDI [43]. Electrical stimulation preceded TMS over the left M1, delivered at an intensity to elicit MEPs of 1 mV, by a time interval based on individual latency of the N20 component of somatosensory evoked potentials (N20 + 2 = SAI Early; N20 + 6 = SAI Late). Similar to CBI and SICI, SAI Early and SAI Late were established as a ratio of 15 CS + TS over 15 TS MEP responses.

### Data analysis

Peak-to-peak MEP amplitudes were measured offline for individual trials. We excluded trials with visible background EMG activity (i.e. activity > 50 μvolts) in the 100 ms preceding the TMS pulse and MEP amplitudes that exceeded three standard deviations around the mean. CBI, SICI and SAI were calculated as the ratio of conditioned responses to the test MEP amplitude. A ratio greater than 1 means there is no inhibitory effect and values close to 0 represent strong inhibitory responses.

Separate repeated measures ANOVA (rmANOVA) were conducted to evaluate changes in CBI, SICI, SAI Early and SAI Late with factors “Time” (Pre, Online, Post) and “Frequency” (5 and 50 Hz). Illustrated data in the figures are expressed as mean and standard error (SE) of the mean, significance value was set as p < 0.05. Where necessary, the Greenhouse–Geisser method was used to correct for violations of sphericity (Mauchly’s tests). When significant main effect or interactions were found, post hoc analysis was conducted with Bonferroni’s multiple comparison test.

## Results

### CBI changes only occur during 5Hz cerebellar tACS

**Fig. 2:**
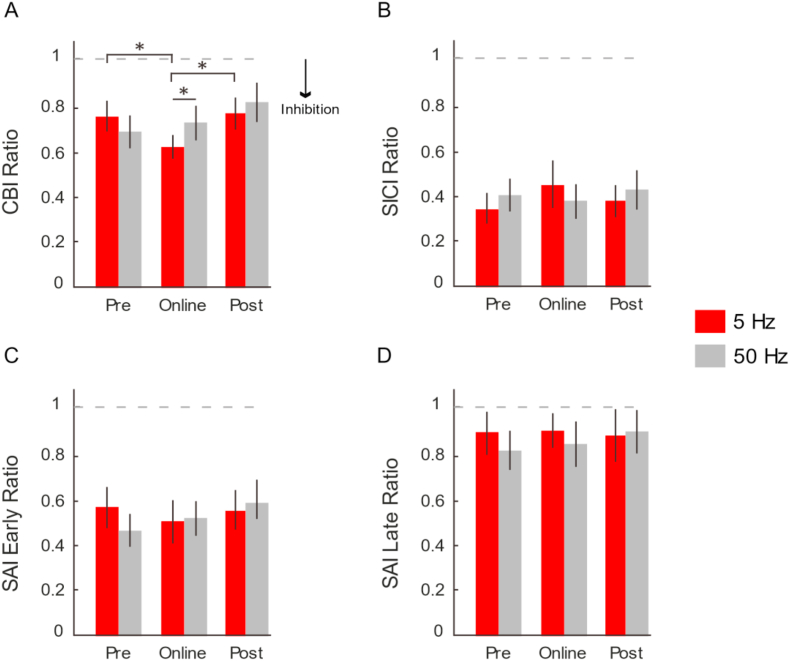
Neurophysiological Responses to cerebellar tACS. Measure of Cerebellar excitability. (A) We compared the responses of CBI prior to (Pre), during (Online) and immediately following (Post) cerebellar tACS at varying frequencies (5Hz = red; 50Hz = grey). We found a significant increase in inhibition only during the application of 5Hz stimulation. Importantly, the levels of CBI prior to and immediately following stimulation remained similar in both 5Hz and 50Hz Data are means ± standard error. (∗) refers to statistically significant changes (p < 0.05). Measures of SICI (B), SAI Early (C) and SAI Late (D). For all measures, there were no significant differences for frequency (5Hz, 50Hz) or time (Pre, Online, Post).

**Fig. 3:**
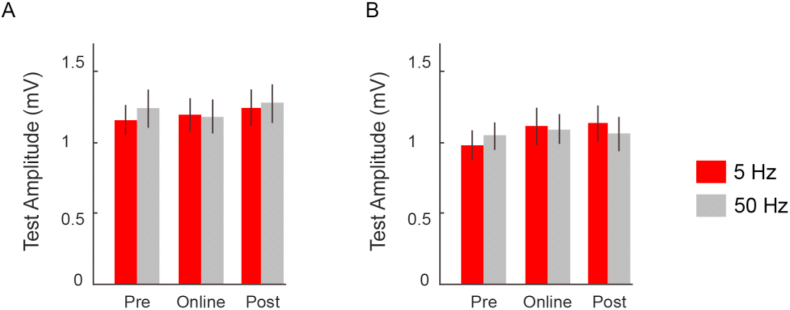
Test MEP amplitudes for CBI/SAI and SICI. Measures of M1 test MEP amplitude. (A) We compared the test MEP amplitudes for CBI and SAI protocols and (B) SICI protocol prior to (Pre), during (Online) and immediately following (Post) cerebellar tACS (5Hz = red; 50Hz = grey). Here, we show that the modulation of CBI following 5Hz stimulation were not due changes in the test response, as we adjusted the stimulator intensity to elicit ∼1 mV test response.

When we compared CBI prior to, during and after the application of cerebellar tACS, we found variable changes depending on the frequency selected (“Time” × “Frequency” interaction: F (2,24) = 4.42; p = 0.023; ηp^2^ = 0.255; Fig. 2a). Specifically, post-hoc comparisons revealed that 5Hz stimulation elicited a stronger CBI during the online application of tACS, when compared to pre (p = 0.030) and post (p = 0.046) stimulation. No such differences between pre-, online- or post-stimulation were found with 50Hz tACS (pre vs online: p > 0.90; pre vs post: p = 0.113; online vs post = 0.761). Moreover, the online change in CBI with 5Hz stimulation was found to be significantly different from that during 50Hz stimulation (*p* = 0.029). This suggests that changes in cerebellar excitability induced by tACS are not only frequency-dependent, but are also sensitive to the online-application of stimulation. In other words, there is no evidence of long-lasting changes that follow entrainment of cerebellar cortex. Importantly, these results are not due to changes in the M1 test stimulus response. The test MEP amplitudes were not different over time or experimental session (“Time” × “Frequency” interaction: F (2,24) = 0.11; p = 0.891; ηp^2^ = 0.006; Fig. 3a), thus our results indicate that changes in CBI are due to the online effects of 5Hz tACS on cerebellar excitability.

### SICI and SAI responses do not change with cerebellar tACS

Previous work had shown that cerebellar-thalamic projections to M1 terminates on inhibitory neurons [10]; thus, changes in cerebellar excitability may lead to modified responses of local inhibitory circuits within M1. However, when we evaluated the SICI responses to cerebellar tACS, rmANOVA showed no significant “Time” × “Frequency” interaction (F (2,24) = 1.307; p = 0.289; ηp^2^ = 0.098; Fig. 2b). Moreover, no changes were found for the test MEP responses that were used to assess SICI (F (2,24) = 0.320; p = 0.729; ηp^2^ = 0.026; Fig. 3b), overall indicating that the observed changes in cerebellar excitability do not result in modulation of inhibitory interneurons in M1.

Similarly, previous work demonstrated that changes in cerebellar excitability influence the response of afferent inputs onto late corticospinal inputs [17]. Thus, we compared early (i.e. processing somatosensory cortex) and late (i.e. proposed to involve the cerebellum) SAI responses to cerebellar tACS. Here, rmANOVA did not reveal a significant “Time” × “Frequency” interaction for both either early (F(2,24) = 0.817; p = 0.454; ηp^2^ = 0.064**;** Fig. 2c) or late SAI (F(2,24) = 1.051; p = 0.365; ηp^2^ = 0.081; Fig. 2d). The summary of all physiological findings (means ± standard error) are given in Table 1. Overall, our results suggest that the effects of tACS are site-specific and that they do not influence how somatosensory inputs are processed in either the cortex or cerebellum.

**Table 1:** Summary of Physiological Findings for each condition of tACS.

	5 Hz tACS			50 Hz tACS		
	Pre	Online	Post	Pre	Online	Post
CBI	0.77 ± 0.07	0.64 ± 0.05	0.78 ± 0.06	0.71 ± 0.07	0.74 ± 0.08	0.82 ± 0.08
SICI	0.35 ± 0.08	0.45 ± 0.10	0.36 ± 0.06	0.40 ± 0.09	0.39 ± 0.07	0.45 ± 0.07
SAI Early	0.55 ± 0.09	0.49 ± 0.08	0.53 ± 0.08	0.46 ± 0.07	0.49 ± 0.07	0.54 ± 0.06
SAI Late	0.88 ± 0.07	0.86 ± 0.09	0.92 ± 0.06	0.81 ± 0.07	0.87 ± 0.06	0.93 ± 0.06

## Discussion

We provided evidence that cerebellar tACS can modulate cerebellar excitability in humans. In particular, we found that theta-band frequency (e.g. 5 Hz) increases the inhibitory tone that the cerebellum exerts over M1. Interestingly, the increase in CBI was found only during the application of the intervention and immediately returned to baseline levels following its termination. This implies that cerebellar tACS does not induce long-term potentiation or depression-like effects, but rather entrains cerebellar activity in a frequency-specific manner.

Previous work has employed techniques such as rTMS and tDCS with the intention to produce long-lasting excitability changes to cerebellar function [18,37,45,46]. These techniques are thought to modulate the activity of Purkinje cells, which exhibit bi-directional plasticity that are critical for fine movement control and learning of motor skills. However, the lack of animal work supporting this logic and the inconsistencies found in the results across several studies suggest that the aftereffects following these interventions are more intricate. On the other hand, our study was designed to investigate whether changes in cerebellar physiology were most prominent during tACS application at a particular frequency. Probing CBI during online rTMS application is not feasible and has only been attempted with tDCS during concurrent muscle contraction [43,47]. Thus, this is the first study to report online changes to cerebellar physiology without the influence of corticospinal activity.

We predicted that theta-frequency stimulation would produce noticeable changes in cerebellar excitability, as various studies have demonstrated that cerebellar granule cells and Golgi cells have a preferential response frequency in the theta-band [33], [34], [35]. For instance, theta-frequency bursting of granule cells dictated are driven by potassium channels, while Golgi cells produce firing frequencies that promote the rhythmic inhibition of granule cells [60]. As such, we propose that cerebellar tACS may produce a stronger CBI effect by increasing the recruitment of cerebellar granule cells activity, whose output axon (i.e. parallel fibre pathways) synapse to the output cells of the cerebellar cortex (i.e. Purkinje cells). Entrainment to tACS may therefore create rhythmic patterns of parallel fibre activity, which in turn would activate inhibitory Purkinje cells. One would therefore expect that tACS increases the efficacy of recruiting parallel fibre-Purkinje cell synapses with TMS, which would increase the amount of CBI.

Why were there no changes in cerebellar excitability found with 50 Hz stimulation? The naturally occurring activity of Purkinje cells is characterized by high-frequency firing that hovers around ∼40–50 Hz [48,49]. We suspect that the entrainment at this frequency neither alters the baseline firing rate of these neurons nor recruits further cerebellar neurons to respond to TMS. In support of this, recent work showed that 50Hz cerebellar tACS did not enhance motor skill learning or retention when compared to sham stimulation [63], thus it could be argued that this frequency is suboptimal for facilitating processes that are involved in motor skill learning. Contrary to this, Naro and colleagues found that 50 Hz tACS reduced CBI and improved the performance of a sequential tapping motor task [27]. It is likely that one key methodological difference could explain the difference in results found here compared to this previous report. Naro and colleagues applied cerebellar tACS for 1 min at 1.0 mA and found CBI changes to occur immediately after stimulation, overall suggesting that an LTD-like mechanism may have reduced the inhibitory output of the cerebellum. Here, we applied tACS with a more intense (1.5 mA) and longer (15 min) stimulation period and found that 50Hz stimulation did not elicit changes in CBI either during or following the protocol. Since our only effect on CBI was found during 5Hz tACS, we argue that entrainment of cerebellar activity is most apparent during stimulation without producing any long-term-like plasticity effects.

It could be argued that only comparing two frequencies presents a limitation to the current findings, as oscillatory activity of the cerebellum is found in various frequency bands [64]. For example, the oscillating local field potentials that occur between 10 and 30 Hz (i.e. beta) have an important functional role in cerebellar-M1 communication during movement preparation and execution [50,51]. A study design with beta-band tACS was originally considered due to its impact to affect cerebellar physiology; however, tACS at this frequency has a higher propensity to elicit phosphenes [52,53]. Applying stimulation at this frequency may therefore disrupt (rather than enhance) the performance of motor and cognitive tasks, ultimately limiting the usefulness of the intervention. Due to this reason, we only considered the physiologically meaningful frequencies of theta- and gamma-bands.

Changes to cerebellar excitability may affect how the brain responds to afferent inputs like SICI and SAI. For example, previous work has shown reduced SICI in the presence of CBI [10], and late-arriving sensory input to the cortex (i.e. late SAI) is modulated by cerebellar tDCS [17,47]. Therefore, one may expect that trans-cerebellar routes (e.g. sensory inputs to the cerebellum and cerebellar projections to M1) may also be modulated by cerebellar tACS. However, we found no evidence of changes to either of these physiological probes, indicating that the effects of cerebellar tACS are limited to the site of stimulation. Cerebellar tACS may still impact how these afferent inputs are processed and may also alter the activity of brain regions connected to the cerebellum, as our results are limited to changes evoked in MEPs recruited with posterior-to-anterior currents over M1. Future studies may consider investigating whether cerebellar tACS differentially modulates distinct cerebellar-cerebral interactions by using directional TMS over M1 (for a review, see Ref. [54]. Additionally, one could implement a combination of TMS and electroencephalogram (EEG) to assess excitability and connectivity following cerebellar tACS [36]. Finally, given the interactions between cerebellar activity and cortical GABA-b inhibitory activity [10,55], future studies should also consider evaluating whether tACS is capable of modulating long intracortical inhibition and cortical silent period.

The lack of a sham-controlled stimulation is potentially another limitation of this study. There is considerable debate, however, as to whether traditional standard sham protocols conducted with alternating current stimulation are effective. For instance, participants exposed to tACS have reported differences in the sensation induced by “sham” and “real” stimulations [56]. This is because the interchanges between phases of stimulation produces visual flickering sensations that last throughout the entire duration of stimulation; therefore, implementing ramp-up and ramp-down strategies, as commonly done for tDCS, might be insufficient for a proper sham control. Moreover, particular frequencies (e.g. alpha and most predominately beta) are more likely to cause retinal phosphenes in participants [65]; therefore, selecting an appropriate sham condition is not trivial. Rather, we argue that having an “active” control condition in which an alternating current is applied for the same duration at frequencies outside of the alpha and beta range would best mimic what subjects feel across sessions. As such, the lack of changes following 50 Hz stimulation to any of our neurophysiological probes makes the case that this frequency can be considered as an “active-sham”, as participants were unaware of differences across sessions. We argue that this strengthens the case that the changes in cerebellar physiology during 5 Hz stimulation are both reliable and robust.

## Conclusion

While the mechanisms underlying the effects of tACS require further investigation from both animal and human research, our results demonstrate the ability of cerebellar tACS to entrain oscillations to specific frequencies. Our approach provides a protocol that is safe and effective for future investigations, which may provide valuable insights for translational studies. For instance, reduced CBI responses are frequently reported in many neurological and psychiatric diseases such as Parkinson’s disease, myoclonus, and schizophrenia [[57], [58], [59]]. Therefore, cerebellar tACS could be used to reset activity levels of the cerebellum, which in turn may have therapeutic benefits in alleviating patient’s symptoms.

## CRediT authorship contribution statement

**Danny Spampinato:** Conceptualization, Methodology, Software, Formal analysis, Investigation, Writing - original draft, Writing - review & editing, Visualization. **Esin Avci:** Formal analysis, Investigation, Writing - original draft, Writing - review & editing. **John Rothwell:** Conceptualization, Resources, Writing - review & editing, Supervision, Funding acquisition. **Lorenzo Rocchi:** Conceptualization, Methodology, Software, Formal analysis, Investigation, Writing - original draft, Writing - review & editing.

## Declaration of competing interest

The authors declare that there is no conflict of interest for this manuscript.
